# The Correlation Analysis between Corneal Biomechanical Properties and the Surgically Induced Corneal High-Order Aberrations after Small Incision Lenticule Extraction and Femtosecond Laser In Situ Keratomileusis

**DOI:** 10.1155/2015/758196

**Published:** 2015-09-21

**Authors:** Wenjing Wu, Yan Wang

**Affiliations:** Tianjin Eye Hospital and Tianjin Eye Institute, Tianjin Ophthalmology and Visual Science Key Laboratory, Clinical College of Ophthalmology, Tianjin Medical University, No. 4, Gansu Road, Heping District, Tianjin 300020, China

## Abstract

*Background*. To investigate the correlation between corneal biomechanics and the surgically induced corneal high-order aberrations (HOAs) after small incision lenticule extraction (SMILE) and femtosecond laser in situ keratomileusis (FS-LASIK). *Methods*. A total of 150 right myopic eyes that underwent SMILE or FS-LASIK surgery were included in this retrospective study, 75 eyes in each group. The corneal hysteresis (CH) and the corneal resistance factor (CRF) with the corneal HOAs of the anterior, posterior, and total cornea were assessed preoperatively and three months postoperatively. Multivariate linear regression was applied to determine the correlations. *Results*. The preoperative CRF was significantly correlated with the induced 3rd–6th-order HOAs and spherical aberration of the anterior surface and the total cornea after SMILE and FS-LASIK surgeries (*P* < 0.05), postoperatively. The CRF was significantly correlated with the induced vertical coma of the anterior and posterior surfaces and the total cornea after SMILE surgery (*P* < 0.05). There was a significant correlation between the CRF and the induced posterior corneal horizontal coma after FS-LASIK surgery (*P* = 0.013). *Conclusions*. The corneal biomechanics affect the surgically induced corneal HOAs after SMILE and FS-LASIK surgery, which may be meaningful for screening the patients preoperatively and optimizing the visual qualities postoperatively.

## 1. Introduction

Currently, laser corneal refractive surgery has become an effective and safe method to correct myopia and astigmatism, especially by use of femtosecond laser in situ keratomileusis (FS-LASIK) and the advanced femtosecond laser small incision lenticule extraction (SMILE) [1, 2]. However, there remain a significant number of corneal high-order aberrations (HOAs) after surgery [3–5], leading to degraded visual qualities and subsequent patient dissatisfaction [6]. Many efforts have been made to investigate the resources of the induced aberrations [3, 4].

Recently, the preoperative corneal biomechanics were identified to play a significant role in the surgically induced astigmatism after cataract surgery [7]. The corneal biomechanics are also closely associated with the corneal HOAs in eyes after intracorneal ring segment implantation [8]. Hence, we suppose that the surgically induced corneal HOAs after SMILE and FS-LASIK surgery may be influenced by the preoperative corneal biomechanics. However, to the best of our knowledge, little study has been done to quantitatively analyze the induced optical changes after SMILE and FS-LASIK surgery from the corneal biomechanical perspective.

Hence, this study was performed to investigate the possible relationships between corneal biomechanical parameters and the surgically induced corneal HOAs in myopic eyes after SMILE and FS-LASIK surgery. Considering these correlations may be important for optimizing the SMILE and FS-LASIK surgeries to achieve satisfactory visual qualities.

## 2. Materials and Methods

### 2.1. Subjects and Examinations

This study enrolled one hundred and fifty eyes of 150 myopic subjects, 75 right eyes in the SMILE group and 75 right eyes in the FS-LASIK group. We retrospectively reviewed the clinical charts of patients who underwent SMILE or FS-LASIK surgery in our department. The inclusion criteria were as follows: subjects who had stable postoperative manifest refraction spherical equivalent (MRSE) within ±0.25 D, together with reliable corneal topographic evaluations and corneal biomechanical examinations preoperatively and 3 months postoperatively. Eyes with postoperative complications such as dry eye or steroid-induced high intraocular pressure were excluded as these complications may influence the corneal high-order aberrations [9, 10]. Seventy-five subjects (38 female/37 male) with the FS-LASIK surgery were included. Patients' age was 24.28 ± 5.24 years (mean ± SD); the range was 18 to 42 years. Spherical correction was −5.22 ± 1.76 D, ranging from −1.50 to −10.50 D, and the cylinder was −0.67 ± 0.52 D, ranging from 0.00 to −2.50 D. Seventy-five subjects (41 female/34 male) who had myopic SMILE refractive surgery were matched for the patient age, sphere, and cylinder. The patients' age was 24.25 ± 5.38 years, ranging from 18 to 41 years. Myopic spherical corrections were −5.12 ± 1.29 D, ranging from −2.25 to −8.50 D. The cylinder was −0.73 ± 0.70 D, ranging from −0.00 to −3.25 D. Detailed clinical data are shown in Table 1. There were no statistically significant differences between the SMILE and FS-LASIK groups in the patients' gender, age, spherical diopter, cylindrical diopter, or MRSE preoperatively.

The research adhered to the tenets of the Declaration of Helsinki and received approval from the Institution Review Board of Tianjin Eye Hospital, Tianjin Medical University. Written informed consent was obtained from all participants after explanations of possible consequences.

Corneal wavefront aberrations were obtained using a rotating Scheimpflug Camera (Pentacam HR, Oculus, Wetzlar, Germany). The Pentacam HR has been used as a noninvasive and reproducible method for the measurement of corneal HOAs of the anterior and posterior surfaces and the total cornea [11, 12]. To avoid misleading effects, the corneas with preoperative scars were excluded from the study, and experienced ophthalmic technicians measured the corneal aberrations of the undilated eyes in low mesopic conditions. In this study, the wavefront aberrations of the anterior corneal surface, the posterior surface, and the total cornea, including the 3rd- to 6th-order HOAs, vertical coma, horizontal coma, and spherical aberration, were analyzed over 6.0 mm central corneal zone with the sign. The qualified readings preoperatively and at 3 months postoperatively were accepted for statistical analysis.

Patients underwent corneal biomechanical examinations through an Ocular Response Analyzer (ORA, Reichert Ophthalmic Instruments; Buffalo, NY, USA), which is the first instrument that could quantify the corneal biomechanical responses clinically [13]. This instrument used an air puff to deform the cornea and generate two principal corneal biomechanical parameters including the corneal hysteresis (CH) and the corneal resistance factor (CRF). CH represents the stiffness along the stromal lamellae; the CRF represents the overall mechanical resistance of the cornea, which is an indicator of the corneal elastic properties [13]. The ORA was used to obtain three or four consecutive measurements in each eye from every patient preoperatively and at 3 months postoperatively. The readings for the qualified examinations with the best waveform score were accepted for statistical analysis.

### 2.2. Surgical Technique

A single experienced surgeon (Yan Wang) performed all the SMILE and FS-LASIK surgeries. The surgical procedure was performed with topical anesthesia using 3 drops of oxybuprocaine hydrochloride (Benoxil, Santen, Inc., Osaka, Japan) applied 3 minutes before surgery. The same femtosecond laser system (VisuMax, Carl Zeiss Meditec AG, Germany) with a 500 kHz repetition rate was used for the SMILE procedure and the FS-LASIK procedure.

The SMILE procedure was performed with the VisuMax femtosecond laser system. The patient's eye was positioned under the interface cone, and the subject was asked to look at the blinking target light. The surgeon adjusted the position of the eye close to the interface cone. Once an appropriate centration (i.e., center of pupil) had been achieved, suction was applied [14–16]. Four cleavage planes were created, including two surfaces of the refractive lenticule, the vertical edge of the refractive lenticule, and a single side-cut incision peripherally of the cornea at the 12 o'clock position. The lenticule was separated from the stromal bed and removed with forceps through the small incision. The lenticule diameter was 6 mm. The optical zone diameter was equal to the lenticule diameter in patients with purely spherical refractive error. If the patient had astigmatism, the software added a transition zone to convert the oval lenticule into a circle. Therefore, the lenticule diameter was 6.0–6.1 mm, depending on the presence or absence of astigmatism [15, 17, 18]. The arc length of the small incision ranged from 2 to 5 mm. The target refraction was within ±0.25 D. The predicted depth of the anterior surface of the lenticule was 110 *μ*m.

The VisuMax system was also used for flap creation in the FS-LASIK procedure; a 400 Hz Allegretto excimer laser system (WaveLight Laser Technologie AG, Germany) was used for stromal ablation with the wavefront-optimized ablation mode. The center of the ablation zone was aligned with the center of the pupil. After stromal ablation, the residual stromal bed was washed with the balanced salt solution, and the flap was repositioned. The target refraction was within ±0.25 D. The intended flap thickness was 100–110 *μ*m with the flap diameters of 7.9–8.0 mm. The hinge of the corneal flap was in the nasal side. All eyes had ablations using an optical zone diameter of 6 mm surrounded by a transition zone of 1.0 mm.

### 2.3. Statistical Analysis

The normality of all data samples was checked with the Kolmogorov-Smirnov test. Differences between the preoperative and postoperative values were compared by the paired *t*-test; differences between SMILE and FS-LASIK were determined by two-tailed independent *t*-test.

Stepwise multivariate linear regression analysis was applied to investigate the determinants of corneal high-order aberrations, using criteria of probability-of-*F*-to-enter ⩽ 0.050 and probability-of-*F*-to-remove ⩾ 0.100. The dependent variable was the changes of the corneal HOAs of the anterior surface, the posterior surface, and the total cornea in SMILE and FS-LASIK, respectively. The explanatory variables include the preoperative CH and the CRF and the manifest refraction spherical equivalent (MRSE), the central corneal thickness (CCT), the intraocular pressure (IOP), and the mean corneal curvature. All regression analysis model assumptions were evaluated by analyzing the condition index in the collinearity diagnostics to exclude the collinearity among the explanatory variables. The Durbin-Watson value was also investigated to confirm the independence of the residual errors. If the condition index was larger than thirty, the last enrolled explanatory variable was excluded, and the other variables were included in the regression analysis. Only modes with the condition index larger than zero and less than thirty and the value of Durbin-Watson tests near two were included. A *P* value less than 0.05 was considered statistically significant. Statistical analysis was performed using SPSS statistical software (version 19.0, Chicago, USA).

## 3. Results

This study was comprised of seventy-five right eyes in the SMILE group and seventy-five right eyes in the FS-LASIK group. Detailed clinical data are shown in Table 1. Preoperatively, there were no statistically significant differences (*P* > 0.05, Student's *t*-test) between the two groups in the patients' gender, age, spherical diopter, cylindrical diopter, manifest refraction spherical equivalent (MRSE), central corneal thickness (CCT), mean corneal curvature, or intraocular pressure (IOP).

### 3.1. Corneal Biomechanics

**Figure 1:**
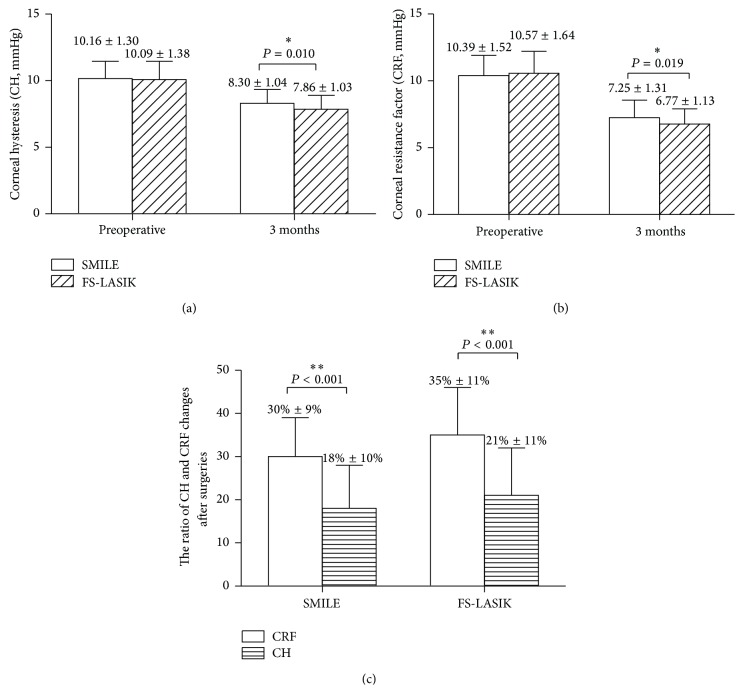
Comparison of the corneal hysteresis (CH) and the corneal resistance factor (CRF) between SMILE and FS-LASIK surgery (a, b); the ratio of CRF changes was significantly higher than that of CH after both the SMILE and the FS-LASIK surgeries (c). The ratio of CRF changes after surgery = ((preoperative CRF − postoperative CRF)/preoperative CRF), ^*∗∗*^ *P* < 0.01, ^*∗*^ *P* < 0.05.

Figure 1 illustrates that the corneal biomechanics significantly decreased after the SMILE (CH: *P* < 0.001, CRF: *P* < 0.001, paired *t*-test) and FS-LASIK (CH: *P* < 0.001, CRF: *P* < 0.001) surgeries. The postoperative CH and the CRF were significantly higher after SMILE than after FS-LASIK (*P* = 0.010, *P* = 0.019; Student's *t*-test, Figures 1(a) and 1(b)).

The CRF decreased 3.14 ± 1.06 mmHg while CH decreased 1.86 ± 1.13 mmHg after the SMILE surgery; the ratio of the CRF changes ((pre-CRF − post-CRF)/pre-CRF) was larger than the reduction ratio of CH (*P* = 0.001, Figure 1(c)) after the SMILE surgery. The CRF decreased 3.80 ± 1.53 mmHg while CH reduced 2.23 ± 1.33 mmHg after the FS-LASIK surgery. The ratio of the CRF changes after the FS-LASIK surgery was also larger than that of CH (*P* = 0.001, Figure 1(c)).

### 3.2. Corneal High-Order Aberrations (HOAs)

**Figure 2:**
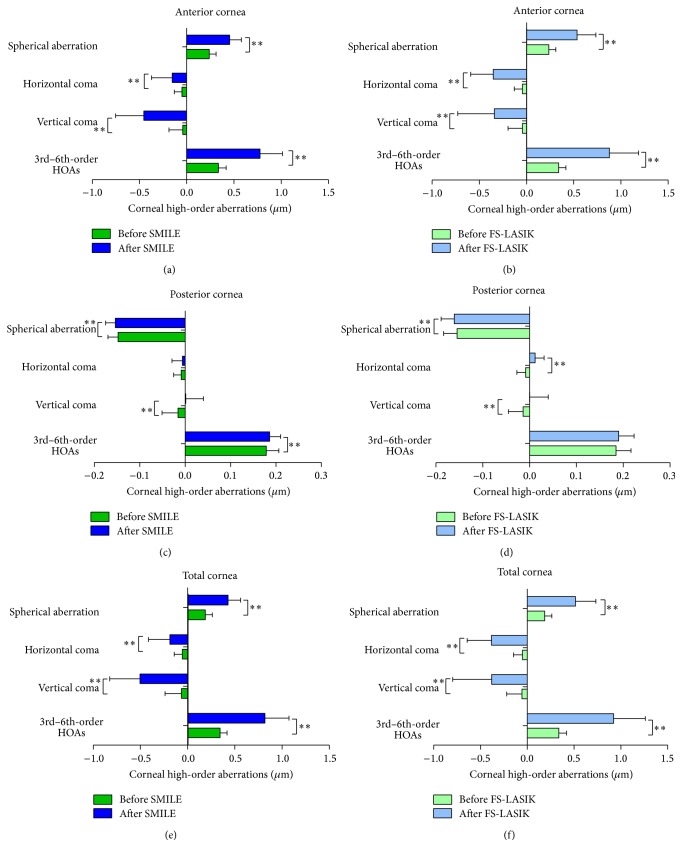
Comparison of the corneal HOAs of the anterior and posterior surfaces and the total cornea after SMILE and FS-LASIK surgeries. ^*∗*^ *P* < 0.05, ^*∗∗*^ *P* < 0.01. HOAs: high-order aberrations.

The corneal HOAs of the anterior surface, the posterior surface, and the total cornea before and after SMILE and FS-LASIK are shown in Figure 2. The 3rd- to 6th-order HOAs and the spherical aberration of the anterior surface and the total cornea exhibit significant increases in both groups (*P* < 0.05).

**Figure 3:**
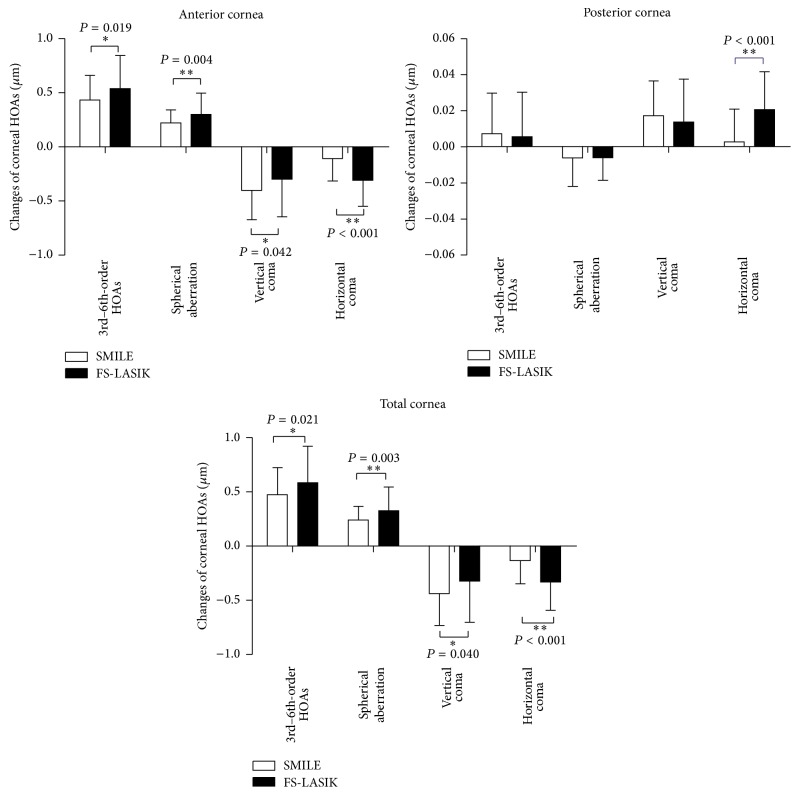
Comparison of the surgically induced corneal high-order aberrations (HOAs) of the anterior surface, the posterior surface, and the total cornea between SMILE and FS-LASIK. ^*∗*^ *P* < 0.05, ^*∗∗*^ *P* < 0.01. HOAs: high-order aberrations.

There were significant differences between SMILE and FS-LASIK groups in the changes of corneal HOAs after surgeries (Figure 3). Specifically, the changes of the 3rd- to 6th-order HOAs, spherical aberration, and horizontal coma of the anterior surface and the total cornea were significantly lower after SMILE surgery than after FS-LASIK surgery (*P* < 0.05, Figure 3). The amounts of the induced vertical coma of the anterior surface and the total cornea were significantly higher after SMILE surgery (*P* = 0.042, *P* = 0.040, Figure 2), whereas the horizontal coma of the anterior cornea and the total cornea was significantly higher after FS-LASIK surgery than SMILE surgery. The induced posterior corneal horizontal coma was significantly lower after SMILE surgery (*P* < 0.001, Figure 3).

### 3.3. Multivariate Analysis of the Relations between Corneal Biomechanics and the Surgically Induced HOAs

**Figure 4:**
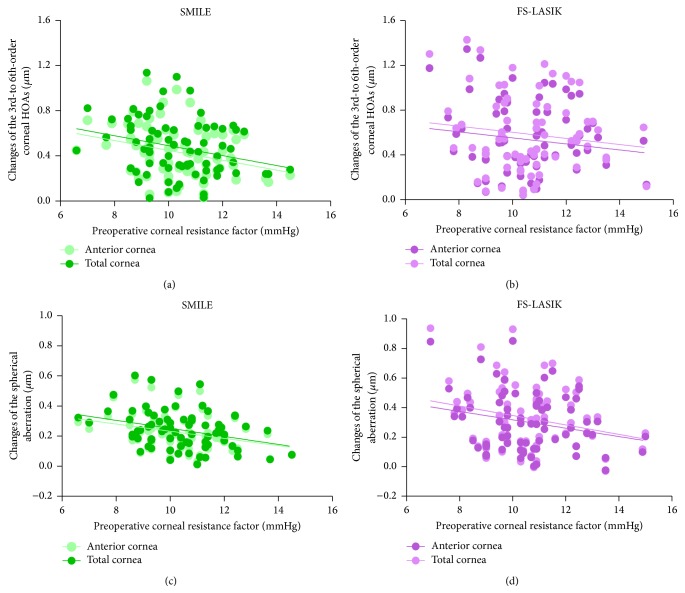
Scatter plots show the significant relations between the preoperative corneal resistance factor (CRF) and the changes in the corneal HOAs for the anterior cornea and the total cornea after SMILE and FS-LASIK surgeries.

**Figure 5:**
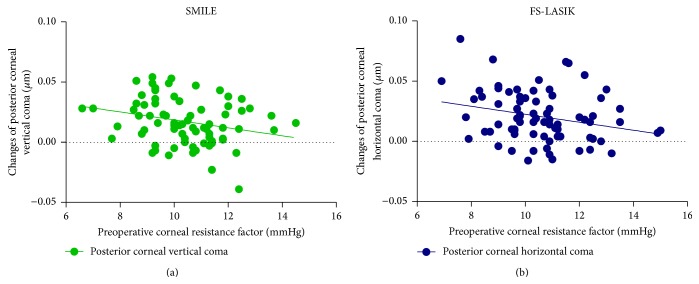
Scatter plots show the significant relations between the preoperative corneal resistance factor (CRF) and the changes of posterior corneal HOAs after SMILE and FS-LASIK surgeries.

The multivariable regression analyses of the surgically induced anterior corneal HOAs, posterior corneal HOAs, and total corneal HOAs are shown in Tables 2, 3, and 4, respectively. After both the SMILE and FS-LASIK procedures, the preoperative CRF showed significantly negative correlations with the induced 3rd- to 6th-order HOAs and the induced spherical aberration of the anterior surface (*P* < 0.01, Figure 4, Table 2) and the total cornea (*P* < 0.01, Figure 4, Table 4). The preoperative CRF also showed significant correlations with the induced vertical coma of the posterior cornea after the SMILE procedure (*P* = 0.024, Table 3, Figure 5). There was a significant correlation between the CRF and the induced horizontal coma of the posterior cornea after FS-LASIK surgery (*P* = 0.013, Table 3, Figure 5).

## 4. Discussion

The present study aims to identify new factors influencing the corneal optical changes after corneal refractive surgeries through corneal biomechanical analysis. We provide evidence that the corneal biomechanics, especially the corneal resistance factor, were significantly correlated with the surgically induced corneal high-order aberrations (HOAs) after the SMILE and FS-LASIK procedures. To the best of our knowledge, this is the first study that investigates the correlations between the CH, the CRF, and the surgically induced corneal HOAs in myopic eyes after SMILE and FS-LASIK surgeries.

We found that the SMILE procedure was superior to the FS-LASIK surgery with respect to the corneal biomechanics. Reinstein et al. [19] and Roy et al. [20] also found better postoperative corneal biomechanics in the SMILE group through the mathematical analyses. Hence, high myopic subjects with lower corneal biomechanics preoperatively are suggested to undergo the SMILE surgery for better postoperative corneal biomechanics. Although some other studies found similar biomechanical results [18, 21] between SMILE and FS-LASIK surgery, the sample size is relative small (30 versus 30, 29 versus 35 eyes). Therefore, other randomized, prospective, contralateral studies with large sample sizes are still needed to confirm our results. Moreover, the present study also found that the CRF was more sensitive to the biomechanical changes after both the SMILE and FS-LASIK surgeries. Piñero et al. [22] also suggest that the CRF has more diagnostic ability than CH for the pathological changes occurring in keratoconic eyes that are characterized as disorganized corneal collagen and weakened corneal biomechanics. This may indicate that the CRF was more sensitive than CH to the optical changes after SMILE and FS-LASIK surgeries.

We also found that the SMILE procedure was superior to FS-LASIK with respect to the corneal high-order aberrations (HOAs), which was in accordance with other studies [23, 24]. The optical advantages of the SMILE procedure may be associated with the flapless surgical technique [5]. These results may be important for those patients with larger pupil size or higher myopia [6, 25]. The SMILE procedure could be performed in these subjects to reduce the optical changes and improve their visual qualities. In addition, we also found the vertical coma was significantly increased after SMILE surgery, whereas the horizontal coma was significantly increased after FS-LASIK surgery. It might result from the superior incision along the vertical axis in SMILE procedure and the nasal-hinge flap in FS-LASIK surgery [5], which may cause imbalanced corneal healing responses and imbalanced optical changes along the axis. Although some studies demonstrated that decentrations [3, 4] play a role in the induction of coma after the SMILE surgery, other studies are still needed to investigate the resources of the vertical coma and horizontal coma to optimize the postoperative visual qualities.

The main finding in the present study is that the preoperative corneal biomechanical parameter CRF was independently correlated with the surgically induced corneal optical changes whatever the spherical equivalent was. These correlations indicated that the lower the CRF, the larger the induced corneal high-order aberrations after refractive surgeries. In other words, corneas with weaker mechanical and structural properties might be linked to more deformable corneal surfaces and larger optical changes of the cornea after surgeries. This might help explain why some subjects are more predisposed to poor visual qualities than others. In terms of correlations between corneal biomechanics and corneal aberrations, previous studies found that the corneal aberrations significantly increased in eyes that underwent radial keratectomy with reduced corneal biomechanical strengths [26, 27]. However, these studies did not investigate the correlations between the preoperative corneal biomechanics and the surgically induced corneal HOAs in eyes after surgery. Recently, Denoyer et al. demonstrated that the corneal biomechanical properties could influence the induced astigmatism in cataract surgery [7]. Piñero et al. also found that the corneal biomechanical properties were correlated with the corneal HOAs after intraocular ring segment implantation [8]. All of these findings demonstrate that the corneal biomechanical properties might be important factors associated with the corneal optical changes after surgeries. Our findings suggest that the clinical surgeons should pay more attention to the corneal biomechanical parameters preoperatively since patients with lower corneal structural properties may suffer from higher corneal HOAs, limiting the potential visual benefits of the SMILE and FS-LASIK surgeries. The corneal biomechanical characteristics might be included in the algorithm of corneal refractive surgeries to optimize the postoperative visual qualities of the SMILE and FS-LASIK surgeries.

There are some limitations in this study. Although this study enrolled 150 subjects, future studies with larger sample size would be needed to confirm our initial observations. Moreover, the present study investigated the correlations between corneal biomechanics and the surgically induced corneal HOAs in the advanced SMILE surgery and the established FS-LASIK surgery. Future studies may demonstrate the possible associations between corneal biomechanics and the surgically induced corneal HOAs in other corneal procedures.

In summary, the changes of corneal high-order aberrations after SMILE and FS-LASIK surgeries were associated with corneal biomechanical properties. Corneal biomechanics may be meaningful for screening patients preoperatively and optimizing the visual qualities postoperatively.

## Figures and Tables

**Table 1:** The characteristics of the subjects in the SMILE and FS-LASIK groups.

Parameters	SMILE (*n* = 75 eyes) Mean ± SD (range)	FS-LASIK (*n* = 75 eyes) Mean ± SD (range)	*P*
Female/male	41/34	38/37	0.744
Age (years)	24.25 ± 5.38 (18 to 41)	24.28 ± 5.24 (18 to 42)	0.959
Sphere (D)	−5.12 ± 1.29 (−2.25 to −8.5)	−5.22 ± 1.76 (−1.50 to −10.50)	0.700
Cylinder (D)	−0.73 ± 0.70 (0 to −3.25)	−0.67 ± 0.52 (0 to −2.5)	0.507
MRSE (D)	−5.49 ± 1.35 (−2.75 to −8.875)	−5.56 ± 1.76 (−1.875 to −11.00)	0.808
CCT (*μ*m)	547.69 ± 27.06 (503 to 620)	545.97 ± 27.71 (500 to 639)	0.701
Km (D)	43.08 ± 1.25 (40.5 to 46.4)	43.32 ± 1.22 (41.2 to 46.0)	0.221
IOP (mmHg)	15.80 ± 2.55 (10.1 to 20.1)	15.79 ± 2.78 (9.4 to 20.7)	0.988

MRSE: manifest refraction spherical equivalent, CCT: central corneal thickness, Km: mean corneal curvature, and IOP: intraocular pressure.

**Table 2:** Stepwise multivariate linear regression analyses of factors that correlated with the alterations of the anterior corneal aberrations after SMILE and FS-LASIK.

Changes of anterior corneal HOAs	SMILE			Adjusted *R* ^2^	FS-LASIK			Adjusted *R* ^2^
	Priority	Beta	*P*		Priority	Beta	*P*	
3rd- to 6th-order HOAs	MRSE	−0.487	<0.001	0.303	MRSE	−0.755	<0.001	0.574
	CRF	−0.305	0.002		CRF	−0.210	0.007	

Spherical aberration	MRSE	−0.451	<0.001	0.279	MRSE	−0.701	<0.001	0.530
	CRF	−0.321	0.002		CRF	−0.299	<0.001	

Vertical coma	MRSE	0.332	0.003	0.135	MRSE	0.302	0.009	0.079
	CRF	0.229	0.037					

Horizontal coma	MRSE	0.237	0.040	0.043	MRSE	0.584	<0.001	0.332

HOAs: high-order aberrations, CRF: corneal resistance factor, MRSE: manifest refraction spherical equivalent, and beta: standardized coefficients.

**Table 3:** Stepwise multivariate linear regression analyses of factors that correlated with the alterations of the posterior corneal aberrations after SMILE and FS-LASIK.

Changes of posterior corneal HOAs	SMILE			Adjusted *R* ^2^	FS-LASIK			Adjusted *R* ^2^
	Priority	Beta	*P*		Priority	Beta	*P*	
Vertical coma	CRF	−0.261	0.024	0.055				0.079

Horizontal coma					CRF	−0.280	0.013	0.107
					MRSE	−0.257	0.023	

HOAs: high-order aberrations, CRF: corneal resistance factor, MRSE: manifest refraction spherical equivalent, and beta: standardized coefficients.

**Table 4:** Stepwise multivariate linear regression analyses of factors that correlated with the alterations of the total corneal aberrations after SMILE and FS-LASIK.

Changes of total corneal HOAs	SMILE			Adjusted *R* ^2^	FS-LASIK			Adjusted *R* ^2^
	Priority	Beta	*P*		Priority	Beta	*P*	
3rd- to 6th-order HOAs	MRSE	−0.496	<0.001	0.298	MRSE	−0.760	<0.001	0.579
	CRF	−0.281	0.005		CRF	−0.200	0.010	

Spherical aberration	MRSE	−0.442	<0.001	0.280	MRSE	−0.697	<0.001	0.526
	CRF	−0.335	0.001		CRF	−0.302	<0.001	

Vertical coma	MRSE	0.338	0.003	0.136	MRSE	0.304	0.008	0.080
	CRF	0.223	0.043					

Horizontal coma	MRSE	0.289	0.012	0.084	MRSE	0.594	<0.001	0.344

HOAs: high-order aberrations, CRF: corneal resistance factor, MRSE: manifest refraction spherical equivalent, and beta: standardized coefficients.
